# A genome assembly of the North American golden eagle, *Aquila chrysaetos canadensis*

**DOI:** 10.1093/jhered/esag022

**Published:** 2026-03-25

**Authors:** Samantha L R Capel, Robert N Fisher, Merly Escalona, Peter H Bloom, Noravit Chumchim, Colin W Fairbairn, Oanh H Nguyen, Ruta M Sahasrabudhe, William E Seligmann, Todd E Katzner, H Bradley Shaffer, Michael R Buchalski

**Affiliations:** Wildlife Genetics Research Unit, Wildlife Health Laboratory, California Department of Fish and Wildlife, Sacramento, CA, United States; Western Ecological Research Center, U.S. Geological Survey, San Diego, CA, United States; Department of Biomolecular Engineering, University of California Santa Cruz, Santa Cruz, CA, United States; Institute for Systems Genomics, University of Connecticut, Stoors, CT, United States; Bloom Biological Inc., Santa Ana, CA, United States; DNA Technologies and Expression Analysis Core Laboratory, Genome Center, University of California-Davis, CA, United States; Department of Ecology and Evolutionary Biology, University of California Santa Cruz, Santa Cruz, CA, United States; DNA Technologies and Expression Analysis Core Laboratory, Genome Center, University of California-Davis, CA, United States; DNA Technologies and Expression Analysis Core Laboratory, Genome Center, University of California-Davis, CA, United States; Department of Ecology and Evolutionary Biology, University of California Santa Cruz, Santa Cruz, CA, United States; Forest and Rangeland Ecosystem Science Center, U.S. Geological Survey, Boise, ID, United States; School of Natural Resources and the Environment, West Virginia University, Morgantown, West Virginia, USA; Department of Ecology and Evolutionary Biology, University of California, Los Angeles, CA, United States; La Kretz Center for California Conservation Science, Institute for Environment and Sustainability, University of California, Los Angeles, CA, United States; Wildlife Genetics Research Unit, Wildlife Health Laboratory, California Department of Fish and Wildlife, Sacramento, CA, United States

**Keywords:** Accipitriformes, *A. chrysaetos canadensis*, California conservation genomics project, CCGP, golden eagle, long-read genome assembly

## Abstract

The golden eagle (*Aquila chrysaetos*) is an apex predator across its Holarctic range. Although chromosome-level reference genome assemblies are available for two of the six golden eagle subspecies (European and Japanese), current assemblies for the North American subspecies (*A. c. canadensis*) were generated using short-read sequencing technology, limiting completeness, contiguity, and accuracy. Here we present a chromosome-length de novo genome assembly for a male *A. c. canadensis* as part of the California Conservation Genomics Project. We used Pacific Biosciences HiFi reads and Omni-C chromatin-proximity sequencing to produce a high-quality assembly consistent with the standard California Conservation Genomics Project reference genome protocol. Our assembly spans 1.28 Gbp and comprises 316 scaffolds with a scaffold N50 of 47.3 Mbp, a contig N50 of 30.3 Mbp, and a benchmarking universal single-copy ortholog completeness score of 97.4%. This reference genome assembly offers a valuable resource for delineating genomic variation and assessing conservation needs in golden eagle populations across California and its North American range more broadly.

## Introduction

**Fig. 1.**
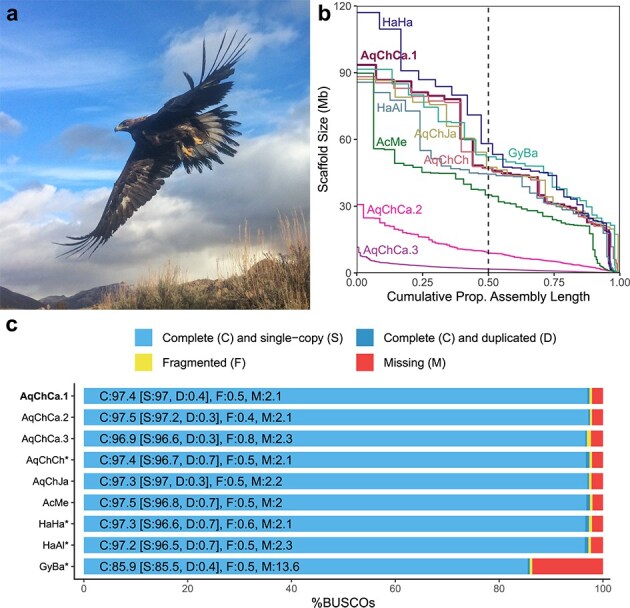
North American golden eagle (*Aquila chrysaetos canadensis*) genome assembly quality comparison. A) A first-year North American golden eagle (photo credit: Peter Bloom). B) Nx plot comparing scaffold size and contiguity of all golden eagle and six other high-quality Accipitridae genome assemblies. Shown is the cumulative proportion of the genome assembly for each scaffold (x axis) plotted against scaffold size (y axis) for each assembly: *A. c. canadensis* (AqChCa.1; North American golden eagle; GCA_030820615.1), *A. c. canadensis* (AqChCa.2; GCA_000766835.1), *A. c. canadensis* (AqChCa.3; GCA_000696035.1), *A. c. chrysaetos* (AqChCh; European golden eagle; GCA_900496995.4), *A. c. japonica* (AqChJa; Japanese golden eagle; GCA_030162315.1), *Haliaeetus albicilla* (HaAl; white-tailed eagle; GCA_947461875.1), *Gypaetus barbatus* (GyBa; bearded vulture; GCA_028022735.1), *Harpia harpyja* (HaHa; harpy eagle; GCA_026419915.1), and *Accipiter melanochlamys* (AcMe; black-mantled goshawk; GCA_027497775.1). Our assembly is shown in bold. C) BUSCO scores for each assembly in reference to the aves_odb10 BUSCO database. Species codes match those of (B), asterisks (*) indicate chromosome-level assemblies, and our assembly is indicated in bold.

As one of the largest raptors in the world, the golden eagle (*Aquila chrysaetos*; Fig. 1a) is a formidable predator found across much of the northern hemisphere (Watson 2010). Golden eagles are found in a variety of habitats (McGahan 1968; Watson 2010), with some individuals exhibiting seasonal migration and others occupying their large home ranges year-round (Poessel et al. 2016, 2022). Although currently listed as a species of least concern (IUCN 2025), there are regional records of decline (Hoffman and Smith 2003; Watson 2010; Katzner et al. 2012) with some populations at serious risk of extirpation (e.g. Ogden et al. 2020), whereas others face substantial anthropogenic mortality (Millsap et al. 2022).

There are currently six recognized subspecies including (1) the European golden eagle (*A. c. chrysaetos*; Linnaeus, 1758) found throughout the northern and central European continent; (2) the Iberian golden eagle (*A. c. homeyeri*; Severtzov, 1888) located in southern Europe, the Mediterranean, and northern Africa; (3) the Asian or Himalayan golden eagle (*A. c. daphanea*; Severtzov, 1888) found throughout mountainous regions of central Asia; (4) the Siberian or Kamchatkan golden eagle (*A. c. kamtschatica*; Severtzov, 1888) found across eastern Russia and northeastern Asia; (5) the Japanese golden eagle (*A. c. japonica*; Severtzov, 1888) located in Japan and Korea; and (6) the North American golden eagle (*A. c. canadensis*; Linnaeus, 1758) found throughout much of North America. As these designations were based on differences in morphology and habitat type (Cramp and Simmons 1980), several studies have investigated the level of genetic support for these lineages. Although mitochondrial haplotypes only identify two lineages separating Mediterranean individuals from all other regions (Nebel et al. 2015), whole-genome resequencing of four of the six subspecies (*A. c. chrysaetos*, *A. c. homeyeri*, *A. c. japonica*, *and A. c. canadensis*) indicates some support for subspecies designations (Sato et al. 2024).

Although high-quality, chromosome-level reference genomes have been generated for the European (GCA_900496995.4; Mead et al. 2021) and Japanese (GCA_030162315.1) subspecies, they are still lacking for many others, including the North American subspecies *A. c. canadensis*. Currently, there are two reference genomes available for *A. c. canadensis* (i.e. GCA_000696035.1 by Doyle et al. 2014 and GCA_000766835.1 by Van Den Bussche et al. 2017). However, these assemblies were generated using short-read sequencing technology that limits assembly contiguity and completeness, and hence the resolution and accuracy, of alignment data (Stephens and Iyer 2018). Although reference genomes of closely related species can be sufficient for aligning population-level data (Galla et al. 2018; Valiente-Mullor et al. 2021), current estimates put divergence of *A. c. canadensis* from the European and Japanese subspecies at ~500 thousand years ago (Kya; Sato et al. 2020). Given sustained allopatry and differences in habitat type (Cramp and Simmons 1980; Sato et al. 2020, 2024), there is a distinct possibility for significant sequence and/or structural variation to have developed. Outstanding questions about the distribution of genomic variation across North American populations could therefore be accurately answered with an updated *A. c. canadensis* reference genome assembly.

Here, we present a chromosome-length reference genome assembly for *A. c. canadensis* as part of the California Conservation Genomics Project (CCGP), a state-funded, collaborative initiative to provide a representative genomic dataset to facilitate regional conservation efforts (Shaffer et al. 2022). We also provide a lift-over gene annotation utilizing the high-quality annotation from the European golden eagle assembly (GCA_900496995.4; Mead et al. 2021). We compare the quality of our assembly to that of the existing North American golden eagle assemblies, as well as six of the highest quality Accipitridae reference genomes, using benchmarking universal single-copy ortholog (BUSCO) completeness scores. As the assembly derives from a male, W-linked sequence is absent. However, all autosomes and the Z chromosome are assembled to chromosome length, with many exhibiting telomere-to-telomere assembly. This reference genome will serve as an important resource for understanding the partitioning of genomic variation among North American golden eagle populations and support management efforts in California and across the expansive range more broadly.

## Methods

### Biological materials

An adult male *A. c. canadensis* was captured in a bow net in Bandy Canyon, San Diego County, California in January 2022 (sample ID: 0679-0282; NCBI BioSample: SAMN35669231). Sample collection was conducted by Dr. Peter Bloom of Bloom Biological Inc. under US Geological Survey (20431) and California Department of Fish and Wildlife (SS-000221) permits. Whole blood was drawn from the brachial vein and stored in an ethylenediaminetetraacetic acid (EDTA)-sodium dodecyl sulfate (SDS) lysis buffer (Rudnick et al. 2005). Following sample collection, the individual was released to the original capture location. Blood was kept at ambient temperature in the field and transferred to a −80°C freezer for long-term storage.

### DNA library preparation and sequencing

#### PacBio HiFi library

High molecular weight genomic DNA (gDNA) was isolated from whole blood using the Sambrook and Russell methodology (Sambrook and Russell 2001) with modifications as first described in (Jain et al. 2018). First, 20 μL of whole blood was added to 2 mL of lysis buffer containing 100 mM NaCl, 10 mM Tris–HCl pH 8.0, 25 mM EDTA, 0.5% (w/v) SDS, and 100 μg/mL Proteinase K. Lysate was incubated at room temperature until the solution was homogenous and then treated with 20 μg/mL RNase A at 37 °C for 30 min. The lysate was cleaned with equal parts of phenol and chloroform using phase lock gels (Quantabio, Beverly, MA; Cat # 2302830). DNA was precipitated by adding 0.4× volume of 5 M ammonium acetate and 3× volume of ice-cold ethanol. The DNA pellet was washed twice with 70% ethanol and resuspended in elution buffer (10 mM Tris, pH 8.0). Purity of gDNA was assessed using NanoDrop ND-1000 spectrophotometer (260/280 ratio = 1.84, 260/230 ratio = 2.36). DNA yield was quantified using Qbit 2.0 Fluorometer (final mass = 33 μg; Thermo Fisher Scientific, Waltham, MA). Integrity of high molecular weight gDNA was verified on a Femto pulse system (Agilent Technologies, Santa Clara, CA) where 95% of DNA had fragment size >100 kilo base pairs (Kbp).

The HiFi SMRTbell library was constructed using the SMRTbell Express Template Prep Kit v2.0 (Pacific Biosciences—PacBio, Menlo Park, CA; Cat. #100-938-900) according to the manufacturer’s instructions. High molecular weight gDNA was sheared to a target size distribution of 15 to 18 Kbp using Diagenode’s Megaruptor 3 system (Diagenode, Belgium; cat. B06010003). The sheared gDNA was concentrated using 0.45X of AMPure PB beads (Pacific Biosciences, Menlo Park, CA; PacBio Cat. #100-265-900) for the removal of single-strand overhangs at 37 °C for 15 min, followed by DNA damage repair at 37 °C for 30 min, end repair and A-tailing at 20 °C for 10 min and 65 °C for 30 min, and finally v3 overhang adapter ligation at 20 °C for 60 min. The SMRTbell library was concentrated with 1X AMPure PB beads, purified via nuclease treatment at 37 °C for 30 min, and size selected using the PippinHT system (Sage Science, Beverly, MA; Cat #HPE7510) to collect fragments > 7 to 9 Kbp (final mean size 15 to 20 Kbp). Finally, the HiFi SMRTbell library was sequenced at the UC Davis DNA Technologies Core (Davis, CA) using one 8M SMRT cell, Sequel IIe sequencing chemistry 2.0, and 30-h movies each on a PacBio Sequel IIe sequencer.

#### Omni-C library

The Omni-C library was prepared using the Dovetail Omni-C Kit (Dovetail Genomics, Scotts Valley, CA) from nucleated blood according to the manufacturer’s protocol with slight modifications. Briefly, chromatin was fixed in place in the nucleus. The fixed chromatin was digested in situ with DNase I. After digestion, the cells were lysed with SDS, and chromatin fragments were bound to chromatin capture beads, digested with DNase I, then extracted. Chromatin ends were repaired and ligated to a biotinylated bridge adapter followed by proximity ligation of adapter-containing ends. After proximity ligation, crosslinks were reversed, and the DNA was isolated from proteins. Purified DNA was treated to remove biotin not internal to ligated fragments. The library was generated using an NEB Ultra II DNA Library Prep kit (New England Biolabs, Ipswich, MA) with an Illumina compatible y-adaptor. Biotin-containing fragments were then captured using streptavidin beads. The post-capture product was split into two replicates prior to PCR enrichment to preserve library complexity with each replicate receiving unique dual indices. The resultant library was quality checked by sequencing at a shallow depth (~2 million total reads) which was fed through Dovetail’s quality control pipeline (https://dovetail-analysis.readthedocs.io/en/latest/whole_genome/qc.html). After passing quality control, the library was sequenced at high depth at the Vincent J. Coates Genomics Sequencing Lab (Berkeley, CA) across two lanes of the Illumina NovaSeq 6000 platform (Illumina, San Diego, CA) to generate ~120 million 2 × 150 bp read pairs.

### Genome assembly

#### Nuclear genome

We performed *A. c. canadensis* genome assembly following the CCGP assembly pipeline v5.0 as outlined in Table 1. Briefly, we first filtered remnant adapter sequences from the PacBio HiFi dataset using HiFiAdapterFilt (Sim et al. 2022). Then, initial diploid-phased assemblies were generated using HiFiasm (Cheng et al. 2022) in HiC mode using the filtered PacBio HiFi reads along with Omni-C short-reads, a process that generates one assembly per haplotype. We then aligned the Omni-C data to each assembly following the Arima Genomics Mapping Pipeline (https://github.com/ArimaGenomics/mapping_pipeline) and scaffolded each assembly using SALSA (Ghurye et al. 2017, 2019).

**Table 1.** Assembly pipeline and software used in preparation of the North American golden eagle (*Aquila chrysaetos canadensis*) genome assembly. Software citations are listed in the text.

Assembly	Software and any non-default options	Version
Filtering PacBio HiFi adapters	HiFiAdapterFilt	Commit 64d1c7b
K-mer counting	Meryl (k = 21)	1
Estimation of genome size and heterozygosity	GenomeScope (-l 23)	2
De novo assembly (contiging)	HiFiasm	0.16.1-r375
	(Hi-C Mode, –primary, output hic.hap1.p_ctg, hic.hap2.p_ctg)	
**Scaffolding**		
Omni-C data alignment	Arima genomics mapping pipeline	Commit 2e74ea4
Omni-C scaffolding	SALSA (-DNASE, -i 20, -p yes)	2
Arima genomics mapping pipeline (AGMP)	BWA-MEM	0.7.17-r1188
	samtools	1.11
	filter_five_end.pl (AGMP)	Commit 2e74ea4
	two_read_bam_combiner.pl (AGMP)	Commit 2e74ea4
	picard	2.27.5
**Omni-C contact map generation**		
Short-read alignment	BWA-MEM (-5SP)	0.7.17-r1188
SAM/BAM processing	samtools	1.11
SAM/BAM filtering	pairtools	0.3.0
Pairs indexing	pairix	0.3.7
Matrix generation	cooler	0.8.10
Matrix balancing	hicExplorer	3.6
	(hicCorrectmatrix correct --filterThreshold -2 4)	
Contact map visualization	HiGlass	2.1.11
	PretextMap	0.1.4
	PretextView	0.1.5
	PretextSnapshot	0.0.3
Manual curation tools	Rapid curation pipeline (Wellcome Trust Sanger Institute, Genome Reference Informatics Team)	Commit 7acf220c
**Genome quality assessment**		
Basic assembly metrics	QUAST (--est-ref-size)	5.0.2
Assembly completeness	BUSCO (-m geno, -l aves)	5.0.0
	Merqury	29 January 2020
	tidk	0.2.0
**Contamination screening**		
Local alignment tool	BLAST+ (-db nt, -outfmt '6 qseqid staxids bitscore std', -max_target_seqs 1, -max_hsps 1, -evalue 1e-25)	2.15
General contamination screening	BlobToolKit	2.3.3
	(HiFi coverage, BUSCO = aves, NCBI Taxa ID = 8962)	
**Mitochondrial assembly**		
Mitochondrial genome assembly	MitoHiFi (-r, -p 90, -o 1, -a animal) Reference: *Aquila chrysaetos* (NCBI:NC_024087.1)	2.2
	MitoFinder (MitoHiFi pipeline parameters)	1.4

The assemblies for each haplotype were manually curated by iteratively generating and analyzing their corresponding Omni-C contact maps. Briefly, to generate the contact maps, we aligned the Omni-C data using BWA-MEM (Li 2013) then identified ligation junctions and generated Omni-C pairs (Lee et al. 2022) using pairtools (Abdennur et al. 2024). Multiresolution Omni-C matrices were generated with cooler (Abdennur and Mirny 2020) and balanced with hicExplorer (Ramírez et al. 2018). We then used HiGlass (Kerpedjiev et al. 2018) and the PretextSuite (https://github.com/sanger-tol/PretextView;  https://github.com/sanger-tol/PretextMap;  https://github.com/sanger-tol/PretextSnapshot) to visualize the contact maps. Misassemblies and misjoins were visualized using HiGlass and PretextView and were identified as abrupt breaks in the intra-scaffold diagonal together with increased inter-scaffold contact. Assemblies were then modified using the Rapid Curation pipeline from the Wellcome Trust Sanger Institute, Genome Reference Informatics Team (https://gitlab.com/wtsi-grit/rapid-curation). Remaining gaps (i.e. joins generated during scaffolding and/or curation) were closed using the PacBio HiFi reads with YAGCloser (https://github.com/merlyescalona/yagcloser). Finally, we checked for contamination using the BlobToolKit Framework (Challis et al. 2020).

#### Mitochondrial genome

We assembled the mitochondrial genome from the PacBio HiFi reads using the reference-guided pipeline MitoHiFi (Allio et al. 2020; Uliano-Silva et al. 2023). The mitochondrial sequence of an existing *A. c. canadensis* (NCBI reference sequence: NC_024087.1) was used as the starting sequence. After nuclear assembly completion, we searched for matches of the resulting mitochondrial assembly sequence in the nuclear genome assembly using BLAST+ (Camacho et al. 2009) and filtered out contigs and scaffolds from the nuclear genome with a percentage of sequence identity >99% and size smaller than the mitochondrial assembly sequence.

### Genome quality evaluation

We generated k-mer counts from the PacBio HiFi reads using meryl (https://github.com/marbl/meryl). The k-mer counts were then used in GenomeScope2.0 (Ranallo-Benavidez et al. 2020) to estimate features including genome size, heterozygosity, and repeat content. We also ran QUAST (Gurevich et al. 2013) to obtain contiguity metrics. To evaluate genome quality and functional completeness, we used BUSCO v5.0.0 (Manni et al. 2021) with the Aves ortholog database (aves_odb10) which contains 8,338 genes. Assessment of base-level accuracy (QV) and k-mer completeness was performed using the previously generated meryl database and merqury (Rhie et al. 2020). We further estimated genome assembly accuracy via BUSCO gene set frameshift analysis using the pipeline described in Korlach et al. (2017). Measurements of phased block size is based on the size of contigs generated by HiFiasm on HiC mode. We follow the quality metric nomenclature established by Rhie et al. 2021, with the genome quality code x.y.P.Q.C where x = log_10_[contig NG50], y = log_10_[scaffold NG50], P = log_10_ [phased block NG50], Q = Phred base accuracy quality value (QV), and C = % genome represented by the first “*n*” scaffolds, following a karyotype of 2*n* = 62 for this species (Nishida-Umehara and Yoshida 1994; Masuda et al. 1998; Challis et al. 2023). Quality metrics for the notation were calculated on the assembly for Haplotype 1 assembly. We further assessed chromosome completeness by identifying telomeric motifs in the largest 27 chromosome-scaffolds by using the telomere identification toolkit tidk v0.2.0 (Brown et al. 2025). We used tidk search to search for the telomeric repeat AACCCT with default window size of 10 Kbp, and the resulting output was plotted using tidk plot.

### Assembly comparisons

To gauge how our assembly compares to other high-quality bird of prey reference genome assemblies, including two other golden eagle subspecies, we compared BUSCO (Manni et al. 2021) completeness scores to the top six currently available Accipitridae assemblies (based on largest scaffold N50 values and lowest number of scaffolds; Supplementary Table S1). This included the European golden eagle (*A. c. chrysaetos*; GCA_900496995.4; Mead et al. 2021), Japanese golden eagle (*A. c. japonica*; GCA_030162315.1), white-tailed eagle (*Haliaeetus albicilla*; GCA_947461875.1; Pálsson et al. 2024), bearded vulture (*Gypaetus barbatus*; GCA_028022735.1; Rhie et al. 2021), harpy eagle (*Harpia harpyja*; GCA_026419915.1; Canesin et al. 2024), and black-mantled goshawk (*Accipiter melanochlamys*; GCA_027497775.1; Catanach and Pirro 2023; all accessed 29 January 2025). We also included the two currently available North American gold eagle reference assemblies (GCA_000696035.1; Doyle et al. 2014 and GCA_000766835.1; Van Den Bussche et al. 2017). We standardized BUSCO scores across assemblies by performing nucleotide-level accuracy assessments on all DNA genome assemblies using aves_odb10.

### Lift-over annotation

We performed lift-over gene annotation utilizing the *A. c. chrysaetos* assembly (GenBank: GCA_900496995.4; Mead et al. 2021) annotation, which was generated using the NCBI Eukaryotic Genome Annotation Pipeline v8.6 (https://github.com/ncbi/egapx). To identify syntenic scaffolds/chromosomes and assess lift-over feasibility, we first aligned our assembly to GCA_900496995.4 using minimap2 v2.29 (options: *-ax* and *asm5*; Li 2018). Syntenic scaffolds were identified by filtering alignments to those with a phred score ≥40 and alignment length ≥50 Kbp. We then performed annotation lift-over using liftoff v1.6.3 (Shumate and Salzberg 2021) with all default options plus *-polish* and *-chroms* where syntenic scaffolds were defined by the filtered minmap2 output. Lift-over gene density was calculated in 1 Mbp windows using bedtools v2.25.0 (Quinlan and Hall 2010) and visualized alongside synteny using circos v0.69 (Krzywinski et al. 2009). Gene annotation completeness was assessed via BUSCO using the aves_odb10 database.

## Results

### Nuclear genome assembly

**Fig. 2.**
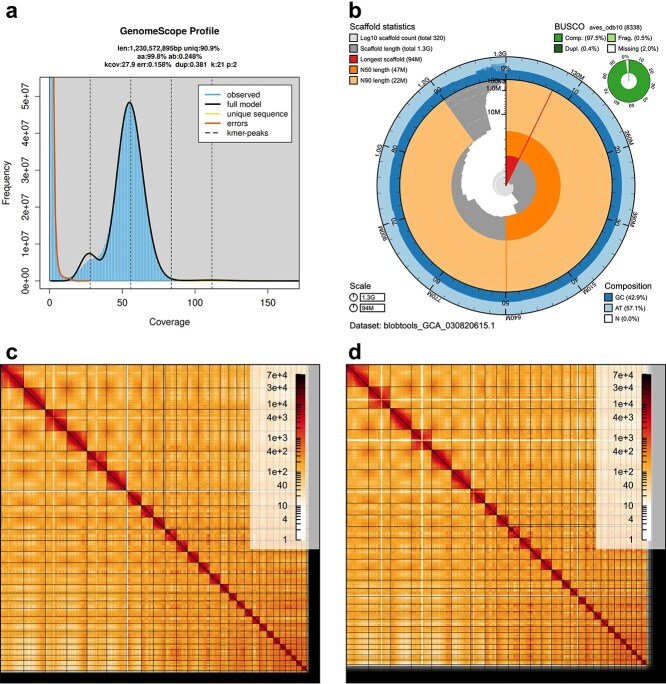
Visualization of assembly metrics for the North American golden eagle (*Aquila chrysaetos canadensis*) genome assembly. A) K-mer spectrum generated using GenomeScope2.0 from PacBio HiFi sequencing data. B) BlobToolKit Snail plot illustrating quality metrics for the primary *A. c. canadensis* assembly (bAquChr2.0.hap1). The plot circumference represents the full length of the assembly. The central red arc and associated line identify the length of the longest scaffold. All other scaffold lengths are shown in dark gray ordered from largest to smallest moving clockwise with lengths indicated by the vertical axis located at 12 o’clock. The central light gray circle shows the cumulative scaffold count using log10 scale. The dark and light orange arcs stipulate the scaffold N50 and N90 values, respectively. The dark to light blue ratios around the perimeter of the circle represent the proportion of AT to GC content at 0.1% length intervals. Omni-C contact maps for C) the primary (i.e., Haplotype 1) and D) alternate (i.e., Haplotype 2) genome assemblies. Contact maps translate the proximity of sequenced regions in 3D space to linear order of sequences. Individual scaffolds are delineated by vertical and horizontal lines and color scales correspond to the number of mapped Omni-C reads.

The Omni-C library generated 154.46 million read pairs and the PacBio HiFi library generated 4.8 million reads. The PacBio HiFi sequences yielded ~55× genome coverage and had an N50 read length of 15,376 bp, a minimum read length of 95 bp, a mean read length of 14,859 bp, and a maximum read length of 60,611 bp (see Supplementary Fig. S1 for read length distribution). Based on the PacBio HiFi data, GenomeScope 2.0 estimated a genome size of 1.23 Gb, a 0.158% sequencing error rate, and 0.248% heterozygosity. The k-mer spectrum shows a bimodal distribution with a major peak at ~55× coverage and a minor peak ~27× coverage, confirming diploidy (Fig. 2a).

The final genome assembly consists of two phased haplotypes (bAquChr2.0.hap1 and bAquChr2.0.hap2). Both assemblies are similar in size, but slightly larger than the estimated genome size from GenomeScope2.0, as has been observed in other taxa (e.g. Pflug et al. 2020). The Haplotype 1 assembly (bAquChr2.0.hap1) consists of 316 scaffolds spanning 1.28 Gbp with a contig N50 of 30.3 Mbp, a scaffold N50 of 47.3 Mbp, a maximum contig size of 92.6 Mbp, and a maximum scaffold size of 93.5 Mbp. The Haplotype 2 assembly (bAquChr2.0.hap2) consists of 284 scaffolds spanning 1.31 Gbp with a contig N50 of 31.6 Mb, a scaffold N50 of 47.6 Mb, a maximum contig size of 87.1 Mbp, and a maximum scaffold size of 90.6 Mbp. Assembly statistics are reported in Table 2 and represented graphically in Fig. 2b and Supplementary  Fig.  S2.

**Table 2.** Sequencing and assembly statistics and accession numbers produced from preparation of the North American golden eagle (*Aquila chrysaetos canadensis*) genome assembly.

**Bio projects and vouchers**	CCGP NCBI BioProject				PRJNA720569		
	Genera NCBI BioProject				PRJNA766266		
	Species NCBI BioProject				PRJNA983307		
	NCBI BioSample				SAMN35669231		
	Specimen identification				0679-02687		
	NCBI Genome accessions			Haplotype 1		Haplotype 2	
	Assembly accession			JAUIRP000000000		JAUIRQ000000000	
	Genome sequences			GCA_030820615.1		GCA_030820595.1	
**Genome sequence**	PacBio HiFi reads	Run	1 PACBIO_SMRT (Sequel II) run: 4.8M spots, 71.1G bases, 42.4 Gb				
		Accession	SRX21418133				
	Omni-C Illumina reads	Run	2 ILLUMINA (Illumina NovaSeq 6000) runs: 154.5M spots, 46.6G bases, 15.9 Gb				
		Accession	SRX21418134,SRX21418135				
**Genome assembly quality metrics**	Assembly identifier (Quality code^a^)		bAquChr2(7.7.P7.Q68.C96)				
	HiFi Read coverage^b^		55.51X				
				**Haplotype 1**		**Haplotype 2**	
	Number of contigs			377		360	
	Contig N50 (bp)			3,02,97,396		3,16,44,863	
	Contig NG50^b^			4,70,19,627		4,59,58,646	
	Longest contigs			9,25,63,760		8,70,68,059	
	Number of scaffolds			316		284	
	Scaffold N50			4,73,18,033		4,76,48,450	
	Scaffold NG50^b^			8,11,72,668		8,40,60,901	
	Largest scaffold			9,35,10,830		9,05,92,526	
	Size of final assembly			1,28,35,23,261		1,31,21,04,294	
	Phased block NG50^b^			4,70,19,627		4,41,83,278	
	Gaps per Gbp (# Gaps)			48(61)		58 (76)	
	Indel QV (Frame shift)			39.77717645		39.76193895	
	Base-pair QV			68.5869		68.5869	
			Full assembly = 68.3582				
	k-mer completeness			97.2028		97.2571	
			Full assembly = 99.82				
	BUSCO completeness (aves) *n* = 8,338		**C**	**S**	**D**	**F**	**M**
		H1^c^	97.40%	97.00%	0.40%	0.50%	2.10%
		H2^c^	97.50%	97.20%	0.30%	0.50%	2.00%
	Organelles	1 Partial mitochondrial sequence				JAUIRP010000316.1	

a Assembly quality code x.y.P.Q.C derived notation, from (Rhie et al. 2021). x = log10[contig NG50]; y = log10[scaffold NG50]; P = log10 [phased block NG50]; Q = Phred base accuracy QV (quality value); C = % genome represented by the first “*n*” scaffolds, following a known karyotype for this species of 2*n* = 62. Quality code for all the assembly denoted by haplotype 1 assembly (bAquChr2.0.hap1).

b Read coverage and NGx statistics have been calculated based on the estimated genome size of 1.23 Gb.

c (H1) Haplotype 1 and (H2) Haplotype 2 assembly values.

During manual curation (see Supplementary Fig. S3 for before-and-after curation contact maps), we made a total of 60 joins (23 on Haplotype 1 and 37 on Haplotype 2) and 9 breaks (4 on Haplotype 1 and 5 on Haplotype 2) based on the Omni-C contact map signal. We closed a total of 4 gaps (1 on Haplotype 1 and 3 on Haplotype 2) and filtered out 1 contig corresponding to mitochondrial contamination. No other contigs were removed or modified. The final Omni-C contact maps show highly contiguous assemblies with 27 chromosome-length scaffolds (Fig. 2c and d**)** per assembly, representative of the 27 known macrochromosomes (Nishida-Umehara and Yoshida 1994; Masuda et al. 1998; Challis et al. 2023). It should be noted, however, that the 4 known microchromosomes have not yet been identified among the remaining scaffolds in either assembly. The genome assembly is available on NCBI GenBank (see Table 2 and Data Availability for details).

### Mitochondrial genome assembly

We assembled a partial mitochondrial genome for *A. c. canadensis*. The final mitochondrial sequence has a size of 18,803 bp, with base composition of A = 25.1%, C = 24.8%, G = 23.4%, T = 26.7%, and consists of nine unique transfer RNAs and two protein coding genes. The mitochondrial genome assembly is available on NCBI GenBank (see Table 2 and Data Availability for details).

### Genome quality evaluation

Haplotype 1 has a BUSCO completeness score for the Aves gene set of 97.4%, a base-pair QV of 68.6, a k-mer completeness of 97.2%, and a frameshift indel QV of 39.8. Haplotype 2 has a BUSCO completeness score for the Aves gene set of 97.5%, a base-pair QV of 68.6, a k-mer completeness of 97.3%, and a frameshift indel QV of 39.8. The Omni-C contact maps show highly contiguous assemblies with at least 27 chromosome-length scaffolds within each haplotype (Fig. 2c and d). Telomere analysis confirmed high levels of completeness among chromosome-sized scaffolds (Sup plementary Figs S4.1 and S4.2). Across both haplotypes, a total of 24 scaffolds (44.4%) show telomeric motifs at both ends (10 on Haplotype 1, 14 on Haplotype 2), 24 (44.4%) have a single telomeric end (14 on Haplotype 1 and 10 on Haplotype 2), and only 6 (11.1%) show missing telomeric motifs at both ends (3 on Haplotype 1, 3 on Haplotype 2). Scaffolds corresponding to each haplotype have been deposited on NCBI (see Table 2 and Data Availability for details).

### Assembly comparisons

Assembly quality is equivalent to that of the top six available Accipitridae assemblies. The largest 31 scaffolds encompass 96.7% of the total assembly size (Fig. 1b). With a known karyotype of 2*n* = 62 (Nishida-Umehara and Yoshida 1994; Masuda et al. 1998), these scaffolds are largely representative of chromosomes. Aves BUSCO completeness scores (Fig. 1c) for the primary haplotype fall among those of the top Accipitridae assemblies (C = 97.4% versus 97.5% for *A. melanochlamys*, 97.4% for *A. c. chrysaetos*, 97.3% for *A. c. japonica*, 97.3% for *H. harpyja*, 97.2% for *H. albicilla*, and 85.9% for *G. barbatus*). In comparison to the other two *A. c. canadensis* assemblies (GCA_000696035.1 and GCA_000766835.1), our assembly had the fewest number of scaffolds (315 versus 42,881 and 1,141, respectively), the largest scaffold N50 (47.3 versus 1.7 and 9.2 Mbp, respectively), the largest contig N50 (30.3 versus 0.016 and 0.172 Mbp, respectively), and a comparable BUSCO completeness score (C = 97.4% versus 96.9% and 97.5%, respectively).

### Lift-over annotation

**Fig. 3.**
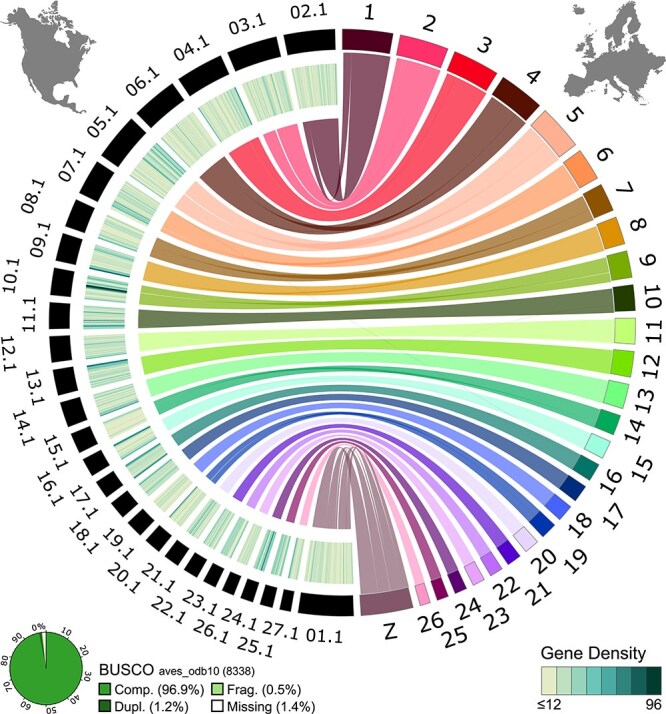
Synteny of the North American golden eagle (*A. c. canadensis*) genome assembly to the current European golden eagle (*A. c. chrysaetos*) reference genome employed for lift-over annotation. The colored rectangles on the right denote assembled chromosomes from the *A. c. chrysaetos* reference genome (bAquChr1.4, GenBank accession: GCA_900496995.4) with chromosome IDs indicated, while black rectangles on the left represent the largest 27 scaffolds from our assembly with labels corresponding to the last 4 characters of the GenBank scaffold name (i.e., JAUIRP0100000XX.X). Synteny is indicated by the colored ribbons which depict the position, length, and orientation of *A. c. canadensis* scaffold to A. c. chrysaetos chromosome alignments. Lift-over gene density is also depicted via the heat map on the inner ring of the *A. c. canadensis* assembly with the respective key shown in the bottom right corner as well as the annotation completeness scores via BUSCO in the bottom left corner.

Alignment of Haplotype 1 to the *A. c. chrysaetos* assembly (GenBank: GCA_900496995.4; Mead et al. 2021) revealed extremely high synteny (Fig. 3) with 96.4% of chromosome-length scaffold sequence (92.4% of total assembly) aligning to 99.2% of chromosomal sequence (96.7% of total assembly) of the *A. c. chrysaetos* assembly after filtering to alignments with Phred scores ≥40 and alignment lengths ≥50 Kbp (Supplementary Table S2). Further, there were very few instances of homologous sequences aligning to different chromosomes. Although over half of the chromosomes (*n* = 15) exhibited one-to-one synteny (e.g. chromosomes 10 to 13), the remaining 12 appear to have large inversions and translocations relative to the *A. c. chrysaetos* assembly as evidenced by twists and crossovers in the synteny plot. These structural differences are especially evident on the Z chromosome. Lift-over annotation was extremely effective, with 1,682,301 chromosomal features (99.9%) successfully aligned, including 20,001 genes (99.8%). Although all genes have primary alignments, 820 (4.10%) have more than one alignment: 6,088 secondary alignments across 714 genes and 246 supplementary alignments across 144 genes (38 genes possess both secondary and supplementary alignments). Performing BUSCO analysis on the annotated gene FASTA revealed the gene annotation as highly complete (C = 98.1% [S = 96.9%, D = 1.2%], F = 0.5%, M = 1.4%; Fig. 3). Finally, gene density visualization (Fig. 3) shows that the distribution of genes across the genome is variable. The lift-over annotation along with accompanying code is available through Dryad (doi:10.5061/dryad.2280gb65r).

## Discussion

We present an updated reference genome for *A. c. canadensis*. The contiguity and synteny to the *A. c. chrysaetos* chromosome-level assembly illustrate that our assembly is equivalent to a chromosome-level assembly. With a base-pair QV of 68.1, contig N50 of 30.3 Mbp, and scaffold N50 of 47.3 Mbp, this assembly exceeds minimum quality targets for the CCGP (Shaffer et al. 2022) based on standards outlined by the Vertebrate Genome Project (Rhie et al. 2021; Shaffer et al. 2022). Given the estimated 500 Kya divergence time of this subspecies (*A. c. canadensis*) from that of the current golden eagle reference genome (*A. c. chrysaetos*; Sato et al. 2020) and apparent structural variation (Fig. 3), this assembly will be a vital resource for genomic studies of North American golden eagles.

Our assembly is a vast improvement on the two previous *A. c. canadensis* assemblies. Although both (GCA_000766835.1 and GCA_000696035.1) exhibit impressive completeness (total size = 1.18 and 1.17 Gbp, BUSCO completeness [C] = 97.5% and 96.9%, respectively), technology has since greatly advanced. Our assembly has vastly improved contiguity, which has major implications for population-level data mapping quality (Stephens and Iyer 2018), and, ultimately, data accuracy. In comparison to the top six Accipitridae reference genomes, our assembly has the largest contig N50 (30.3 versus 21.9 Mbp for *A. c. chrysaetos*, 18.1 Mbp for *G. barbatus*, 17.7 Mbp for *A. melanochlamys*, 16.9 Mbp for *A. c. japonica*, 16.8 Mbp for *H. harpyja*, 4.6 Mbp for *H. albicilla*) and has both a scaffold N50 (47.3 Mbp; 58.4 Mbp for *H. harpyja*, 52.2 Mbp for *G. barbatus*, 47.5 Mbp for *A. c. japonica*, 46.9 Mbp for *A. c. chrysaetos*, 44.4 Mbp for *H. albicilla*, and 35.0 Mbp for *A. melanochlamys*) and a BUSCO completeness score (Fig. 1c) in-line with all other genomes. Moreover, the majority of chromosome-sized scaffolds (88.9%) contain telomeric motifs, further emphasizing assembly completeness.

Assessment of synteny to the *A. c. chrysaetos* assembly (GCA_900496995.4; Mead et al. 2021) suggested high support for utilizing the existing gene annotation for lift-over annotation (Fig. 3). Of note are a number of large inversions and translocations in our *A. c. canadensis* Haplotype 1 assembly relative to the *A. c. chrysaetos* assembly, with the highest number of inversions located on the Z chromosome scaffold (JAUIRP010000001.1). However, a number of inversions coincide with gaps in the contact map (Fig. 2c). Given the quality of both the HiFi and Omni-C sequencing data, these could be true structural variants, but more detailed analyses are required to rule out the possibility of misassemblies, which is beyond the scope of this version of the assembly. If real, the structural variation exhibited between these subspecies indicates significant genomic divergence which, in turn, may have implications for adaptive differentiation or even reproductive incompatibilities (Wellenreuther et al. 2019; Mérot et al. 2020), further emphasizing the importance of this updated, high-quality *A. c. canadensis* assembly.

Although our assembly is highly complete, it should be noted that the four known microchromosomes for this species (Masuda et al. 1998) have not been identified among our 316 scaffolds. Nor have they been assembled for any previous golden eagle reference genome. Microchromosomes are found in nearly all bird species and tend to be highly conserved (Waters et al. 2021). Currently, there are a number of avian reference genomes with annotated microchromosomes including both model (e.g. *Gallus gallus*; GRCg7b) and non-model species (e.g. *H. albicilla* [GCA_947461875.1; Pálsson et al. 2024]*, H. harpyja* [GCA_026419915.1; Canesin et al. 2024]*, A. melanochlamys* [GCA_027497775.1; Catanach and Pirro 2023]), some of which were incorporated into our assembly comparisons. Identification and complete assembly of microchromosomes are currently beyond the scope of this assembly version due to the standardized nature of the CCGP genome assembly pipeline. Although historically challenging, microchromosomes can be assembled in a targeted fashion either during assembly using specialized tools like MicroFinder (Mathers et al. 2025) or after assembly by aligning unplaced scaffolds to a closely related genome with assembled microchromosomes using long-read aligners like minimap2 (Li 2018). However, even with these tools, successful microchromosomes assembly may still prove elusive due to the high difficulty of manual assembly curation of short sequence fragments and/or significant karyotype differences from reference datasets (O’Connor et al. 2019). Though small in size, bird microchromosomes are typically gene-dense with a mean of 40 genes per Mbp compared with 17 genes per Mbp in macrochromosomes (Waters et al. 2021) and thus likely play important biological roles. Hence, microchromosomes should be considered in future golden eagle assembly versions and avian reference genome assemblies, generally.

As part of the CCGP initiative to provide a comprehensive regional genomic dataset, this reference genome can aid in delineating genomic variation of the North American golden eagle. Subsequent work that makes use of the vast collection of high-quality genomic resources provided by the CCGP are likely to prove as powerful examples of how genomic data may support and improve conservation management. For example, this assembly can facilitate answering questions related to population structure, patterns of inbreeding, and local adaptation in the western United States where year-round residents are concentrated (Cornell Lab of Ornithology 2025). Understanding the partitioning of North American golden eagle populations, how they are connected, and their evolutionary relationship to the landscape could prove crucial for managing and conserving this apex predator and, in turn, the habitats they occupy.

## Supplementary Material

REVISED_Supplemental_Tables_and_Figures_esag022

## Data Availability

Data generated for this study are available under NCBI BioProject PRJNA983307. Raw sequencing data for sample 0679-02687 (NCBI BioSample SAMN35669231) are deposited in the NCBI Short Read Archive (SRA) under SRR25693257 for the PacBio HiFi sequencing data, and SRR25693255-56 for the Omni-C Illumina sequencing data. GenBank accessions for Haplotype 1 and Haplotype 2 genome sequences are GCA_030820615.1 and GCA_030820595.1, and the assembly accessions are JAUIRP000000000 and JAUIRQ000000000, respectively. The GenBank accession for the mitochondrial genome assembly is JAUIRP010000316.1. Assembly scripts and other data for the analyses presented can be found on GitHub at www.github.com/ccgproject/ccgp_assembly. Additional code and data are available through Dryad (doi:10.5061/dryad.2280gb65r).
