# Supplementary material for: A genome assembly of the North American golden eagle, Aquila chrysaetos canadensis
Source: J Hered. 2026 Mar 25;117(4):945–56. doi: 10.1093/jhered/esag022 (PMC13326411; doi:10.1093/jhered/esag022)
Supplement: REVISED_Supplemental_Tables_and_Figures_esag022 [file revised_supplemental_tables_and_figures_esag022.docx]

# Supplemental Tables & Figures

**Table S1.** Additional high-quality Accipitridae assemblies for quality comparison to the new North American golden eagle (*Aquila chrysaetos canadensis*) genome assembly.

| **Assembly Name** | **GenBank ID** | **Year** | **Common Name** | **Species** | **Assembly Code** | **Size (Gbp)** | **Scaff N50 (Mbp)** | **Contig N50 (Mbp)** | **# Scaffs** |
| --- | --- | --- | --- | --- | --- | --- | --- | --- | --- |
| Aquila_chrysaetos-1.0.2^[1]^ | GCA_000766835.1 | 2014 | Golden Eagle (North American) | *Aquila chrysaetos canadensis* | AqChCa.2 | 1.180 | 9.2 | 0.172 | 1,141 |
| AquilaChrysaetos1^[2]^ | GCA_000696035.1 | 2014 | Golden Eagle (North American) | *Aquila chrysaetos canadensis* | AqChCa.3 | 1.171 | 1.7 | 0.016 | 42,881 |
| bAquChr1.4^[3]^ | GCA_900496995.4 | 2021 | Golden Eagle (European) | *Aquila chrysaetos chrysaetos* | AqChCh | 1.234 | 46.9 | 21.9 | 144 |
| Ajapo_1 | GCA_030162315.1 | 2023 | Golden Eagle (Japanese) | *Aquila chrysaetos japonica* | AqChJa | 1.188 | 47.5 | 16.9 | 156 |
| bHalAlb1.1^[4]^ | GCA_947461875.1 | 2022 | White-tailed Eagle | *Haliaeetus albicilla* | HaAl | 1.320 | 44.4 | 4.6 | 188 |
| bGypBar2.pri^[5]^ | GCA_028022735.1 | 2023 | Bearded vulture | *Gypaetus barbatus* | GyBa | 1.365 | 52.2 | 18.1 | 232 |
| bHarHar1_primary_haplotype^[6]^ | GCA_026419915.1 | 2022 | Harpy Eagle | *Harpia harpyja* | HaHa | 1.351 | 58.1 | 16.8 | 322 |
| ASM2749777v1^[7]^ | GCA_027497775.1 | 2022 | Black-mantled Goshawk | *Accipiter melanochlamys* | AcMe | 1.398 | 35.0 | 17.7 | 449 |
| 1 Van Den Bussche R.A., Judkins M.E., Montague M.J., Warren W.C. 2017. A Resource of Genome-Wide Single Nucleotide Polymorphisms (SNPs) for the Conservation and Management of Golden Eagles. Journal of Raptor Research. 51:368–377.  2 Doyle J.M., Katzner T.E., Bloom P.H., Ji Y., Wijayawardena B.K., DeWoody J.A. 2014. The Genome Sequence of a Widespread Apex Predator, the Golden Eagle (Aquila chrysaetos). PLoS One. 9:e95599.  3 Mead D., Ogden R., Meredith A., Peniche G., *et al*. 2021. The genome sequence of the European golden eagle, Aquila chrysaetos chrysaetos Linnaeus 1758. Wellcome Open Res. 6:112.  4 Pálsson S., Skarphéðinsson K.H., Heintz J., Quarfordt P., *et al*. 2024. The genome sequence of the white-tailed eagle, Haliaeetus albicilla (Linnaeus, 1758). Wellcome Open Res. 9:575.  5 Rhie A., McCarthy S.A., Fedrigo O., Damas J., *et al*. 2021. Towards complete and error-free genome assemblies of all vertebrate species. Nature. 592:737–746.  6 Canesin L.E.C., Vilaça S.T., Oliveira R.R.M., Al-Ajli F., *et al*. 2024. A reference genome for the Harpy Eagle reveals steady demographic decline and chromosomal rearrangements in the origin of Accipitriformes. Sci Rep. 14:19925.  7 Catanach T.A., Pirro S. 2023. The Complete Genome Sequences of 87 Species of Hawks (Accipitriformes, Aves). Biodiversity Genomes. | | | | | | | | | |

**Table S2.** Alignment results for synteny assessment between the North American golden eagle (*Aquila chrysaetos canadensis*; “Query”) and European golden eagle (*Aquila chrysaetos chrysaetos*; “Target”) genome assemblies corresponding to **Figure 3**. Alignment coordinates (“Start” and “End” positions) are 0-based.

| **Query Scaffold Name** | **Query Scaffold Length** | **Query Alignment Start** | **Query Alignment End** | **Alignment Direction** | **Target Scaffold Name** | **Target Scaffold Length** | **Target Alignment Start** | **Target Alignment End** | **# Residue Matches** | **Alignment Block Length** | **Mapping Quality** |
| --- | --- | --- | --- | --- | --- | --- | --- | --- | --- | --- | --- |
| JAUIRP010000002.1 | 86719258 | 1009987 | 15780273 | + | NC_044004.1 | 85456622 | 116 | 14791619 | 13946915 | 14862916 | 60 |
| JAUIRP010000002.1 | 86719258 | 15966678 | 86650895 | + | NC_044004.1 | 85456622 | 14963028 | 85456578 | 67457981 | 70940991 | 60 |
| JAUIRP010000003.1 | 86091380 | 4 | 31030190 | - | NC_044005.1 | 83001952 | 50730950 | 81789998 | 29806430 | 31101244 | 60 |
| JAUIRP010000003.1 | 86091380 | 30957685 | 31242544 | - | NC_044008.1 | 76621501 | 37284126 | 37531289 | 144606 | 345554 | 60 |
| JAUIRP010000003.1 | 86091380 | 34874284 | 74104744 | - | NC_044005.1 | 83001952 | 11465630 | 50726008 | 38034396 | 39299441 | 60 |
| JAUIRP010000003.1 | 86091380 | 74312012 | 85577756 | - | NC_044005.1 | 83001952 | 7 | 11278392 | 10663478 | 11332553 | 60 |
| JAUIRP010000004.1 | 81172668 | 153776 | 57791691 | - | NC_044006.1 | 79382107 | 22181067 | 79382100 | 55190074 | 57726261 | 60 |
| JAUIRP010000004.1 | 81172668 | 58207166 | 58706991 | + | NC_044006.1 | 79382107 | 21666956 | 22166930 | 491993 | 500011 | 60 |
| JAUIRP010000004.1 | 81172668 | 58707017 | 63927263 | - | NC_044006.1 | 79382107 | 16500654 | 21666942 | 4994236 | 5256210 | 60 |
| JAUIRP010000004.1 | 81172668 | 63917195 | 63972967 | - | NC_044006.1 | 79382107 | 16500654 | 16556470 | 51650 | 55869 | 60 |
| JAUIRP010000004.1 | 81172668 | 64005460 | 64056898 | + | NC_044006.1 | 79382107 | 16500654 | 16556470 | 45402 | 56470 | 60 |
| JAUIRP010000004.1 | 81172668 | 64051735 | 64102591 | + | NC_044006.1 | 79382107 | 16500654 | 16556470 | 45292 | 55892 | 60 |
| JAUIRP010000004.1 | 81172668 | 64097428 | 64148389 | + | NC_044006.1 | 79382107 | 16500654 | 16556470 | 45245 | 55998 | 60 |
| JAUIRP010000004.1 | 81172668 | 64207744 | 80668953 | - | NC_044006.1 | 79382107 | 41 | 16357277 | 15713332 | 16524342 | 60 |
| JAUIRP010000005.1 | 79619711 | 32574 | 37345263 | + | NC_044008.1 | 76621501 | 41 | 37260543 | 35527799 | 37478090 | 60 |
| JAUIRP010000005.1 | 79619711 | 39992692 | 79515348 | + | NC_044008.1 | 76621501 | 37311904 | 76621488 | 37883348 | 39671491 | 60 |
| JAUIRP010000006.1 | 78089343 | 676 | 837738 | - | NC_044007.1 | 77266225 | 6 | 841825 | 787333 | 843790 | 60 |
| JAUIRP010000006.1 | 78089343 | 1144101 | 53869447 | + | NC_044007.1 | 77266225 | 982963 | 53612893 | 50176232 | 52899131 | 60 |
| JAUIRP010000006.1 | 78089343 | 54030630 | 77899224 | + | NC_044007.1 | 77266225 | 53396424 | 77257280 | 22716346 | 23948046 | 60 |
| JAUIRP010000007.1 | 60365236 | 5106091 | 14822316 | - | NC_044009.1 | 54402963 | 0 | 9702871 | 9332711 | 9725121 | 60 |
| JAUIRP010000007.1 | 60365236 | 15553329 | 60268903 | + | NC_044009.1 | 54402963 | 9724922 | 54402954 | 43204594 | 44777154 | 60 |
| JAUIRP010000007.1 | 60365236 | 60310649 | 60361211 | - | NC_044019.1 | 30610376 | 31 | 61648 | 17338 | 73280 | 42 |
| JAUIRP010000008.1 | 48288136 | 217650 | 27786016 | + | NC_044010.1 | 47779391 | 6 | 27609615 | 26264720 | 27668856 | 60 |
| JAUIRP010000008.1 | 48288136 | 27830649 | 48151641 | + | NC_044010.1 | 47779391 | 27434093 | 47779358 | 19393111 | 20378128 | 60 |
| JAUIRP010000009.1 | 47318033 | 114526 | 6484116 | + | NC_044011.1 | 46934974 | 18 | 6379895 | 6083453 | 6396760 | 60 |
| JAUIRP010000009.1 | 47318033 | 6653405 | 6805557 | + | NC_044011.1 | 46934974 | 6550185 | 6672131 | 96908 | 153276 | 60 |
| JAUIRP010000009.1 | 47318033 | 6868974 | 46966060 | + | NC_044011.1 | 46934974 | 6797201 | 46934723 | 38900550 | 40210627 | 60 |
| JAUIRP010000010.1 | 46003966 | 135852 | 25510618 | + | NC_044012.1 | 45241448 | 10 | 25384426 | 24538192 | 25452540 | 60 |
| JAUIRP010000010.1 | 46003966 | 25510763 | 26989464 | - | NC_044012.1 | 45241448 | 25778402 | 27389455 | 1449609 | 1613472 | 60 |
| JAUIRP010000010.1 | 46003966 | 26994267 | 27502198 | + | NC_044012.1 | 45241448 | 25389041 | 25803684 | 406117 | 508314 | 60 |
| JAUIRP010000010.1 | 46003966 | 28440742 | 28550744 | - | NC_044012.1 | 45241448 | 27395919 | 27505920 | 104224 | 110035 | 60 |
| JAUIRP010000010.1 | 46003966 | 28860307 | 45910481 | + | NC_044012.1 | 45241448 | 28196353 | 45241432 | 16451316 | 17078913 | 60 |
| JAUIRP010000011.1 | 44804728 | 278287 | 44733586 | - | NC_044013.1 | 43949808 | 26 | 43949716 | 42338288 | 44541484 | 60 |
| JAUIRP010000012.1 | 44123374 | 405438 | 984179 | - | NC_044014.1 | 43755789 | 43175469 | 43755749 | 484321 | 599176 | 60 |
| JAUIRP010000012.1 | 44123374 | 1005139 | 1364728 | - | NC_044014.1 | 43755789 | 42711191 | 43071783 | 333963 | 365169 | 60 |
| JAUIRP010000012.1 | 44123374 | 1859727 | 43996876 | - | NC_044014.1 | 43755789 | 4 | 41978543 | 40535737 | 42193731 | 60 |
| JAUIRP010000013.1 | 43796735 | 68482 | 43657719 | - | NC_044015.1 | 43481500 | 8 | 43481456 | 41915216 | 43681195 | 60 |
| JAUIRP010000014.1 | 43057329 | 49258 | 42389343 | - | NC_044016.1 | 41790845 | 2144 | 41790832 | 40205434 | 42441604 | 60 |
| JAUIRP010000015.1 | 34930673 | 215935 | 25987143 | + | NC_044017.1 | 34337559 | 26 | 25767152 | 24428981 | 25859140 | 60 |
| JAUIRP010000015.1 | 34930673 | 26280302 | 34861195 | + | NC_044017.1 | 34337559 | 25767670 | 34335476 | 8142587 | 8593738 | 60 |
| JAUIRP010000016.1 | 31724257 | 173091 | 27898021 | + | NC_044018.1 | 30976502 | 15 | 27733702 | 26299119 | 27794151 | 60 |
| JAUIRP010000016.1 | 31724257 | 28118502 | 29241539 | + | NC_044018.1 | 30976502 | 27868868 | 28609205 | 669344 | 1139818 | 60 |
| JAUIRP010000016.1 | 31724257 | 29378974 | 31624636 | + | NC_044018.1 | 30976502 | 28748105 | 30976416 | 2114232 | 2278905 | 60 |
| JAUIRP010000017.1 | 31065923 | 70560 | 21965523 | - | NC_044019.1 | 30610376 | 8803687 | 30602545 | 20807252 | 21942446 | 60 |
| JAUIRP010000017.1 | 31065923 | 22206429 | 25302356 | - | NC_044019.1 | 30610376 | 5564041 | 8657093 | 2993777 | 3098032 | 60 |
| JAUIRP010000017.1 | 31065923 | 25488771 | 31060621 | + | NC_044019.1 | 30610376 | 31 | 5578654 | 5341415 | 5596983 | 60 |
| JAUIRP010000018.1 | 30793200 | 654144 | 30404211 | - | NC_044020.1 | 29699254 | 10740 | 29699223 | 28483356 | 29818697 | 60 |
| JAUIRP010000019.1 | 29841911 | 299682 | 28882665 | + | NC_044021.1 | 28555659 | 16 | 28555576 | 27282595 | 28617980 | 60 |
| JAUIRP010000020.1 | 29234236 | 6090 | 12865910 | + | NC_044022.1 | 27976474 | 664 | 12863996 | 12203798 | 12921281 | 60 |
| JAUIRP010000020.1 | 29234236 | 13953718 | 29118915 | + | NC_044022.1 | 27976474 | 12859453 | 27976462 | 14270938 | 15191654 | 60 |
| JAUIRP010000021.1 | 27030575 | 255778 | 20704889 | - | NC_044023.1 | 25309643 | 4836466 | 25309608 | 19715444 | 20485369 | 60 |
| JAUIRP010000021.1 | 27030575 | 20852067 | 21143908 | - | NC_044023.1 | 25309643 | 4430533 | 4722480 | 280819 | 292152 | 60 |
| JAUIRP010000021.1 | 27030575 | 21271156 | 25569409 | - | NC_044023.1 | 25309643 | 3605 | 4302403 | 4127795 | 4305494 | 60 |
| JAUIRP010000022.1 | 25439910 | 275859 | 25123979 | + | NC_044024.1 | 24756580 | 6 | 24756574 | 23749371 | 24895756 | 60 |
| JAUIRP010000023.1 | 22753485 | 5958 | 22528117 | + | NC_044025.1 | 22514005 | 48127 | 22494087 | 21519608 | 22558777 | 60 |
| JAUIRP010000024.1 | 22006387 | 167747 | 21935969 | + | NC_044027.1 | 20993271 | 11 | 20993261 | 20245101 | 21800260 | 60 |
| JAUIRP010000025.1 | 21748050 | 3 | 56254 | - | NC_044013.1 | 43949808 | 30524601 | 30580834 | 51565 | 56452 | 60 |
| JAUIRP010000025.1 | 21748050 | 1157947 | 21073908 | - | NC_044028.1 | 19844373 | 11 | 19844357 | 19002817 | 19956078 | 60 |
| JAUIRP010000026.1 | 21155479 | 92435 | 9017925 | + | NC_044026.1 | 21012167 | 12 | 8919873 | 8537711 | 8939979 | 60 |
| JAUIRP010000026.1 | 21155479 | 9022177 | 9092015 | - | NC_044026.1 | 21012167 | 8651716 | 8725398 | 22871 | 75037 | 60 |
| JAUIRP010000026.1 | 21155479 | 9092294 | 9263270 | + | NC_044026.1 | 21012167 | 8669434 | 8844790 | 129512 | 176001 | 60 |
| JAUIRP010000026.1 | 21155479 | 9205754 | 21100599 | + | NC_044026.1 | 21012167 | 9101550 | 21012120 | 11299503 | 11920241 | 60 |
| JAUIRP010000026.1 | 21155479 | 21095868 | 21155469 | - | NC_044022.1 | 27976474 | 15197 | 66200 | 15445 | 62043 | 60 |
| JAUIRP010000027.1 | 19163776 | 953609 | 994271 | + | NC_044029.1 | 17717355 | 17646775 | 17714609 | 32026 | 67922 | 50 |
| JAUIRP010000027.1 | 19163776 | 986331 | 1066531 | + | NC_044029.1 | 17717355 | 17561995 | 17609595 | 16252 | 80852 | 60 |
| JAUIRP010000027.1 | 19163776 | 1192412 | 1380722 | - | NC_044029.1 | 17717355 | 17561995 | 17717346 | 75165 | 206681 | 60 |
| JAUIRP010000027.1 | 19163776 | 1372782 | 19108661 | - | NC_044029.1 | 17717355 | 6033 | 17714609 | 16898521 | 17792027 | 60 |
| JAUIRP010000001.1 | 93510830 | 1178695 | 11105412 | + | NC_044030.1 | 88216475 | 17 | 8958527 | 8455239 | 9958133 | 60 |
| JAUIRP010000001.1 | 93510830 | 10864310 | 11512768 | - | NC_044030.1 | 88216475 | 8335086 | 8958528 | 394520 | 715174 | 60 |
| JAUIRP010000001.1 | 93510830 | 11407375 | 32050491 | + | NC_044030.1 | 88216475 | 9259968 | 29519744 | 18587152 | 20793577 | 60 |
| JAUIRP010000001.1 | 93510830 | 34199603 | 38820602 | + | NC_044030.1 | 88216475 | 29514327 | 34095929 | 4269334 | 4674159 | 60 |
| JAUIRP010000001.1 | 93510830 | 39170668 | 61408443 | + | NC_044030.1 | 88216475 | 34487861 | 56587751 | 20713324 | 22356102 | 60 |
| JAUIRP010000001.1 | 93510830 | 61606445 | 61666872 | + | NC_044030.1 | 88216475 | 56874581 | 56923328 | 33686 | 60486 | 60 |
| JAUIRP010000001.1 | 93510830 | 61654090 | 93190062 | + | NC_044030.1 | 88216475 | 56874581 | 88214443 | 29884554 | 31604532 | 60 |
| JAUIRP010000001.1 | 93510830 | 93443961 | 93503012 | - | NC_044022.1 | 27976474 | 664 | 60085 | 22569 | 61820 | 60 |


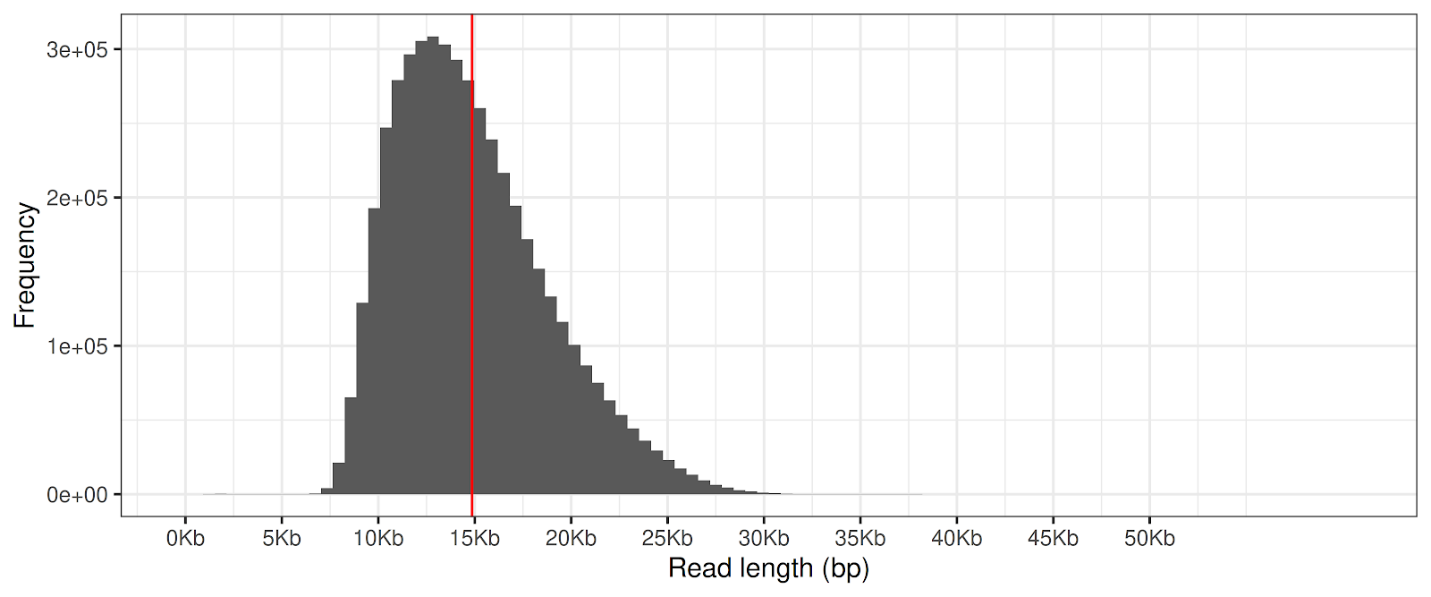


**Fig. S1.** PacBio HiFi raw sequencing read lengths for the North American golden eagle (*Aquila chrysaetos canadensis*) genome assembly. The red vertical line represents average read length.


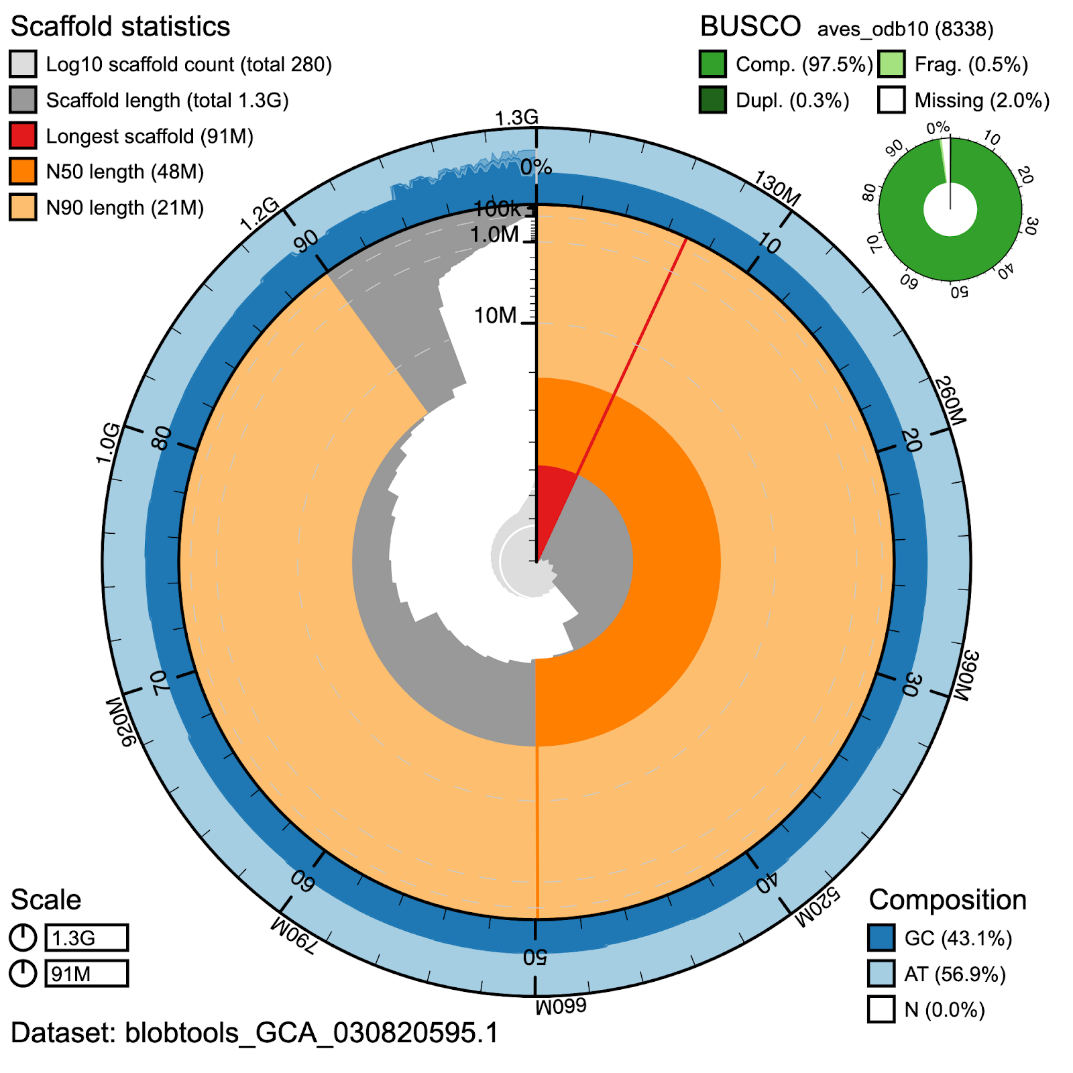


**Fig. S2.** BlobToolKit Snail plot illustrating quality metrics for the alternate *Aquila chrysaetos canadensis* assembly (bAquChr2.0.hap2). The plot circumference represents the full length of the assembly. The central red arc and associated line identify the length of the longest scaffold. All other scaffold lengths are shown in dark gray ordered from largest to smallest moving clockwise with lengths indicated by the vertical axis located at 12 o’clock. The central light gray circle shows the cumulative scaffold count using log10 scale. The dark and light orange arcs stipulate the scaffold N50 and N90 values, respectively. The dark to light blue ratios around the perimeter of the circle represent the proportion of AT to GC content at 0.1% length intervals.

|  | Before curation | After curation |
| --- | --- | --- |
| Haplotype 1 | 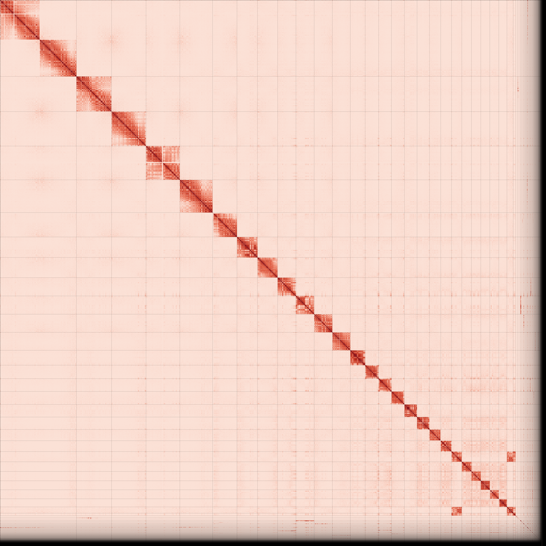 | 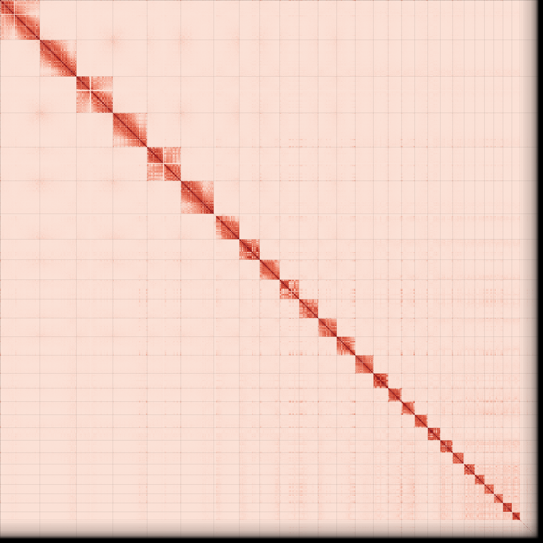 |
| Haplotype 2 | 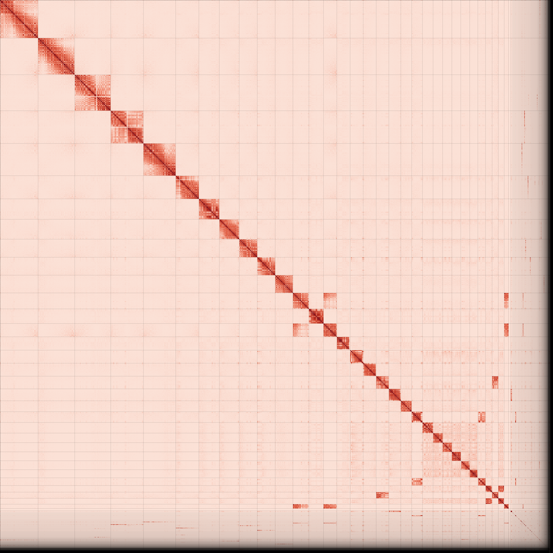 | 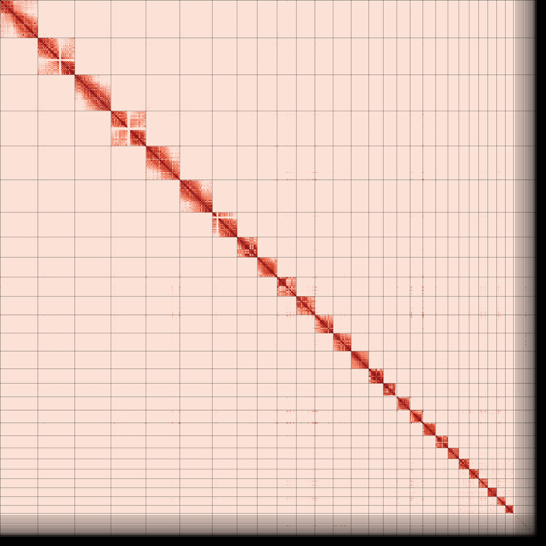 |

**Fig. S3**. Curation process. Before-and-after contact maps showing the edits done per haplotype during the curation process.


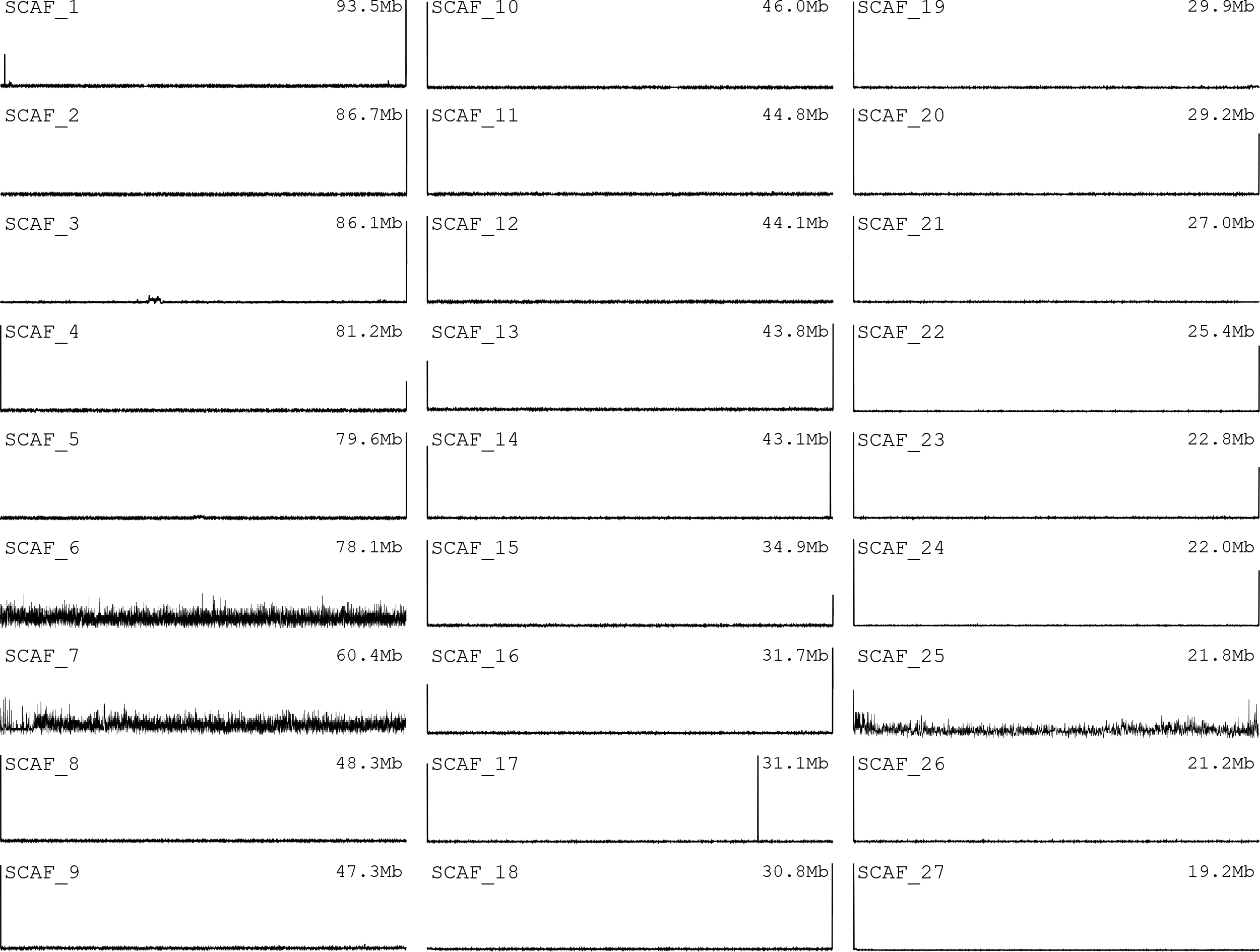


**Fig. S4.1**. Telomere identification for Haplotype 1. Shown here is the output of the telomere identification toolkit. Each plot shows the position (horizontal axis) and frequency (vertical axis) of telomeric motifs for the 27 chromosome-sized scaffolds in the assembly.


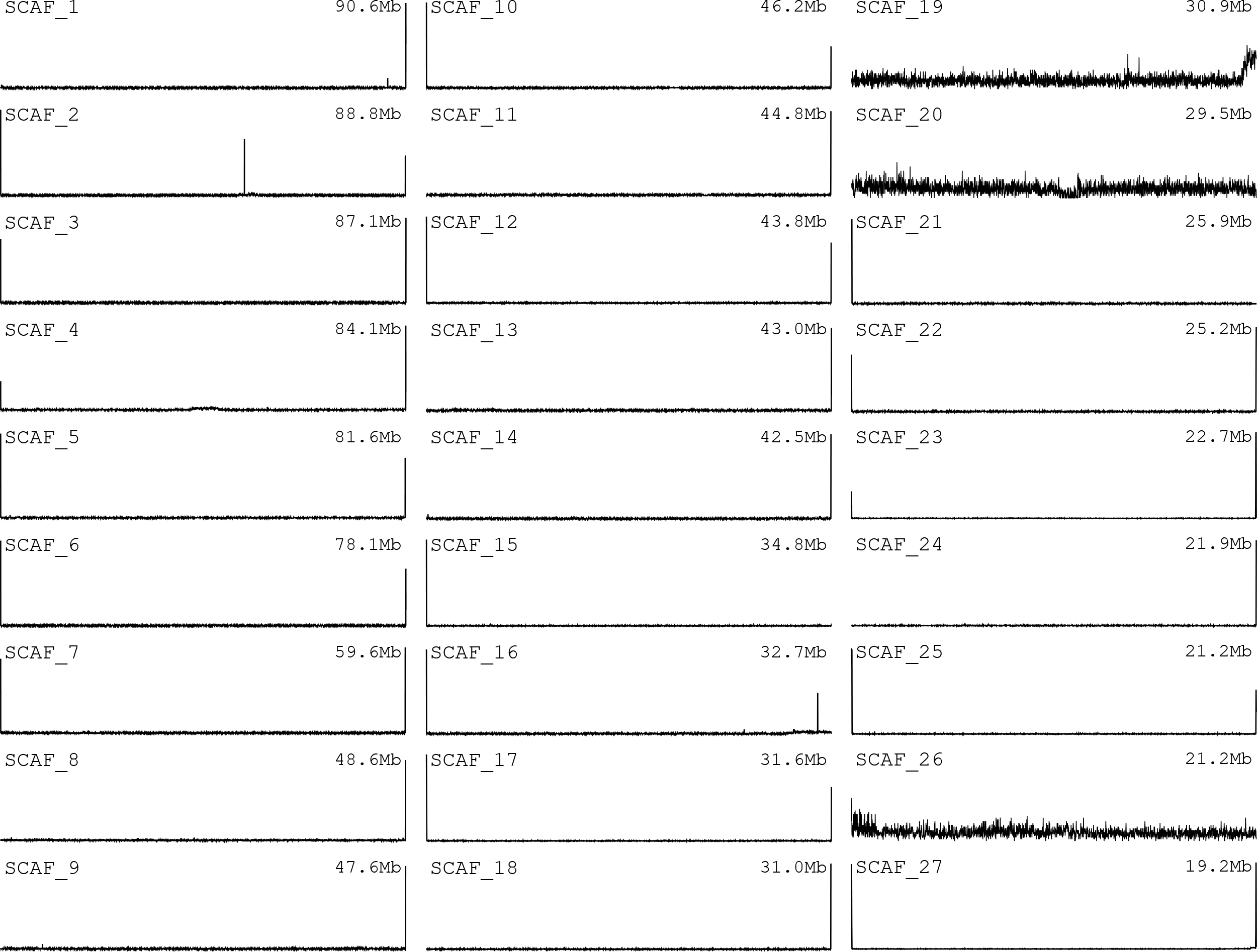


**Fig. S4.2**. Telomere identification for Haplotype 2. Shown here is the output of the telomere identification toolkit. Each plot shows the position (horizontal axis) and frequency (vertical axis) of telomeric motifs for the 27 chromosome-sized scaffolds in the assembly.
